# Quantifying the influence of Bessel beams on image quality in optical coherence tomography

**DOI:** 10.1038/srep23483

**Published:** 2016-03-24

**Authors:** Andrea Curatolo, Peter R. T. Munro, Dirk Lorenser, Parvathy Sreekumar, C. Christian Singe, Brendan F. Kennedy, David D. Sampson

**Affiliations:** 1 Optical+Biomedical Engineering Laboratory, School of Electrical, Electronic & Computer Engineering, The University of Western Australia, 35 Stirling Highway, Crawley WA 6009, Australia; 2 Centre for Microscopy, Characterisation & Analysis, The University of Western Australia, 35 Stirling Highway, Crawley, WA 6009, Australia

## Abstract

Light scattered by turbid tissue is known to degrade optical coherence tomography (OCT) image contrast progressively with depth. Bessel beams have been proposed as an alternative to Gaussian beams to image deeper into turbid tissue. However, studies of turbid tissue comparing the image quality for different beam types are lacking. We present such a study, using numerically simulated beams and experimental OCT images formed by Bessel or Gaussian beams illuminating phantoms with optical properties spanning a range typical of soft tissue. We demonstrate that, for a given scattering parameter, the higher the scattering anisotropy the lower the OCT contrast, regardless of the beam type. When focusing both beams at the same depth in the sample, we show that, at focus and for equal input power and resolution, imaging with the Gaussian beam suffers less reduction of contrast. This suggests that, whilst Bessel beams offer extended depth of field in a single depth scan, for low numerical aperture (NA < 0.1) and typical soft tissue properties (scattering coefficient, *μ*_*s*_ = 3.7 mm^−1^ and high scattering anisotropy, *g* > 0.95), superior contrast (by up to ~40%) may be obtained over an extended depth range by a Gaussian beam combined with dynamic focusing.

Scattering is the basis of OCT contrast in turbid tissue^1^,^2^,^3^, yet it progressively degrades the image quality with depth^4^,^5^,^6^,^7^, causing unacceptable degradation often before the back-scattered signal reduces to the system noise floor. In the absence of absorption, the most important factors in determining this degradation are the imaging depth in the tissue, *z*_*0*_, the scattering coefficient, *μ*_*s*_, and scattering anisotropy, *g* 6,8,9. Thus far, studies of their effects on OCT image quality have been confined to the use of Gaussian beams and, although several of these have analysed the effect of scattering anisotropy^10^,^11^, most have studied the impact of only the scattering coefficient^4^,^5^,^12^,^13^ at various depths.

Bessel beams have been proposed and demonstrated for use with OCT to extend the depth of field (DOF) over that of a Gaussian beam, whilst preserving the transverse resolution^14^,^15^,^16^. An extended DOF is particularly attractive in Fourier-domain OCT, which captures a whole depth scan in a single detector dwell time without axial scanning. One drawback of the Bessel beam is that, in order to maintain a more axially uniform irradiance than the Gaussian beam, energy is necessarily distributed more broadly transverse to the optical axis. As a consequence, the on-axis irradiance is everywhere below that of the peak Gaussian irradiance, for beams of equal power, thereby causing an inherent SNR penalty in comparison to the Gaussian beam at focus^16^. At the same time, however, in a low-scattering sample, the Bessel beam maintains a higher axial irradiance than the Gaussian beam in the out-of-focus region beneath the focus, i.e., it gains an SNR advantage, owing to its extended DOF. For this latter reason, Bessel beams have been used to improve imaging through turbid media, including tissue^14^,^17^,^18^, and the explanation of their performance has been linked to their self-reconstructing property. The self-reconstructing property^19^,^20^ is stated as follows. The amplitude profile of the Bessel beam is largely restored to its original form after a characteristic propagation distance, if part of the beam has been obstructed or distorted.

Rigorous analysis of OCT imaging performance in turbid media with Bessel or Gaussian beams has not been reported. Thus, whilst some comparison of OCT images acquired with Bessel or Gaussian beams (or Bessel beam illumination and Gaussian beam detection) has been made^14^,^15^,^16^, definitive testing of the apparent advantages of the self-reconstructing property of a Bessel beam, beyond the simple case of partial transverse beam occlusion^17^, has not been done. The choice of optimal beam type for OCT imaging in turbid tissue requires such an analysis. We report on the first rigorous computational and experimental analysis of the influence of Bessel and Gaussian beams on OCT contrast in turbid media. Our analysis contains a number of novel elements, including use of a sophisticated full-wave electromagnetic simulation, benchmarked against experiment, a novel test phantom containing well-defined structure covering a wide range of spatial frequencies, and probing the effects of a range of scattering anisotropies. Our analysis allows us to draw firm conclusions regarding the use of Bessel and Gaussian beams in OCT.

The basic setup, depicted schematically in Fig. 1, comprises a Gaussian or Bessel beam (NA = 0.12) passing through a thin scattering overlayer to image a target. The beams have the same input power and the centre of their respective DOFs is located at the same depth in the sample. We used three scattering overlayers, each with the same scattering coefficient (*μ*_s_ = 3.7 mm^−1^) chosen to be typical of tissue^21^, but with different scattering anisotropy (*g* = 0.94–0.99) and a fourth scatterer-free (SF) overlayer for comparison. The narrow range of high scattering anisotropies was chosen to be representative of tissue and to highlight the high sensitivity to this parameter in imaging in turbid media^22^. We used two imaging targets: one comprising random point scatterers to image the system point spread function (PSF) versus depth, termed the PSF phantom, and another comprising pillars of varying sizes to measure the OCT contrast of differently sized features, termed the contrast phantom.

The separation of the highly forward scattering layer and imaging target helps to isolate the effects on image quality of image-degrading contributions (arising from the layer) from image-forming contributions (arising from the target)^23^, according to the presence or absence of scattering in the overlayer.

We seek to assess image quality for Gaussian and Bessel beams versus scattering anisotropy in terms of OCT contrast. We define OCT contrast^24^ as the difference in dB between the square of the average OCT signal amplitude, 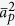, in a homogeneous area of a pillar in the contrast phantom, and the square of the average OCT signal amplitude, 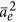, in a homogeneous area around the pillar, near the border between the two:


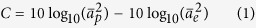


We carried out the assessment of OCT contrast in three steps. We firstly experimentally confirmed that neither the PSF phantom nor the contrast phantom alter the measured degradation due to the overlayer. Secondly, we analysed how the PSF generated using either beam is affected by scattering from the overlayers and, thirdly, we showed experimentally where OCT contrast is better maintained in turbid tissue with either beam.

The first step is achieved by fitting analytic models^25^,^26^,^27^,^28^ to the experimental peak and ensemble-averaged OCT signal intensity versus depth to validate the predominance of single scattering in our imaging targets. In practice, this means that we expect the PSF phantom not to add any OCT signal attenuation, and the contrast phantom to produce a very low attenuation outside the pillars, compatible with single-scattering model fits^29^.

Secondly, to test the degradation of the PSF in the presence of tissue-like scattering, we simulate how the illumination irradiance of either beam type is distorted after propagating through the overlayer. We use ensemble-averaged computational results to quantify the PSF degradation. We also experimentally image the PSF phantom, with and without scattering in the overlayers, for a qualitative comparison.

The PSF degradation due to the overlayers is produced by the forward-scattered background (image-degrading component)^30^ coherently added^31^ to the diffraction-limited illumination beam (image-carrying component), the irradiance of which is simply the scattering-free illumination irradiance attenuated by 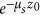. We quantify this degradation by calculating the ratio of the ideally attenuated diffraction-limited irradiance peak and the background at a particular axial location, i.e., the on-axis signal-to-background ratio (SBR)^5^,^6^,^32^,^33^. We, thus, evaluate the SBR as a function of depth along the optical axis for both beams and all overlayers.

Thirdly, with the PSF degradation, there is an associated OCT contrast degradation. To show the link between reduced SBR and OCT contrast, and to compare the contrast performances of both beams, we use the contrast phantom and experimentally analyse the pillar-to-embedding casting OCT contrast, *C*, for a range of overlayer anisotropies.

Novel rigorous electromagnetic (EM) simulations of the two beam types, propagating through the scattering overlayers into the PSF phantom, were used to assess the SBR. Experiments with a dual beam OCT system were carried out on characterization of the imaging targets, on the experimental validation of the simulation, and on the OCT contrast reduction.

## Results

### Signal-to-Background Ratio

**Figure 2:**
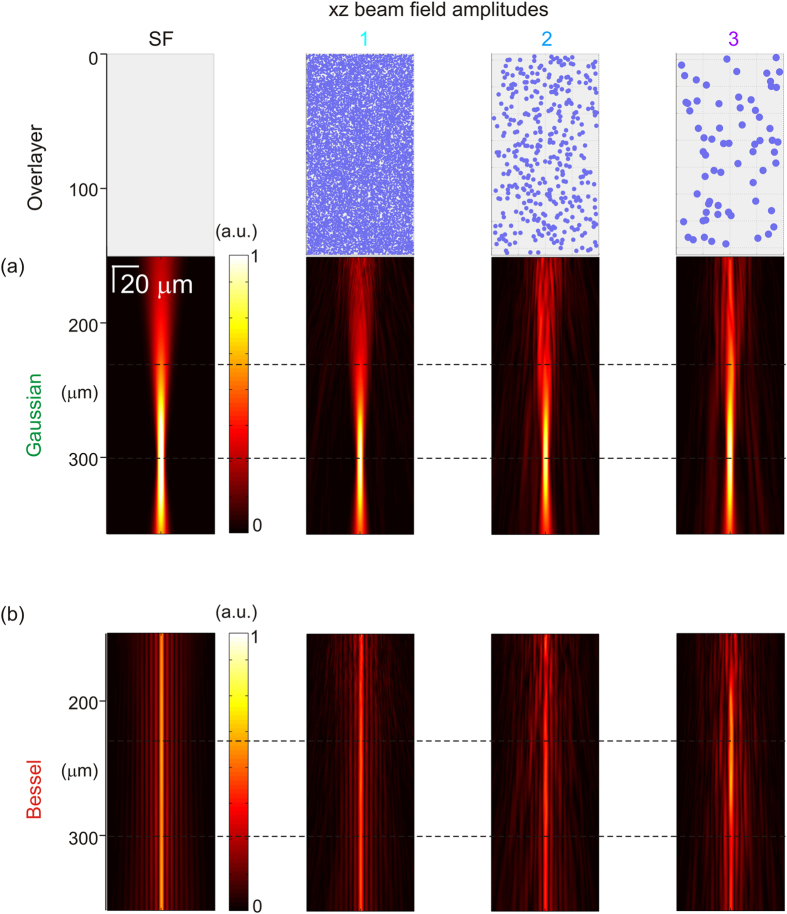
Simulated beam profiles after propagation through Overlayers 1–3 with increasing anisotropy from left to right compared to (left) propagation through a scattering-free (SF) overlayer. Degradation of a single beam profile versus propagation (DOF centre at 300 microns) for (**a**) Gaussian, and (**b**) Bessel beams on a linear amplitude scale, where 1 a.u. represents in each case the peak Gaussian field amplitude at focus after attenuation by the overlayer. Dashed horizontal lines in (a) and (b) show the locations of beam profiles presented in Fig. 3.

Figure 2 presents the simulated beam field amplitude after propagating through each of the overlayers. (The linear field amplitude scale is directly comparable to that of Supplementary Fig. S1). The results in Fig. 2 show a significant increase in image-degrading background around the optical axis with increasing anisotropy, even though all three scattering overlayers equally attenuate the diffraction-limited peak of the illumination beam.

**Figure 3:**
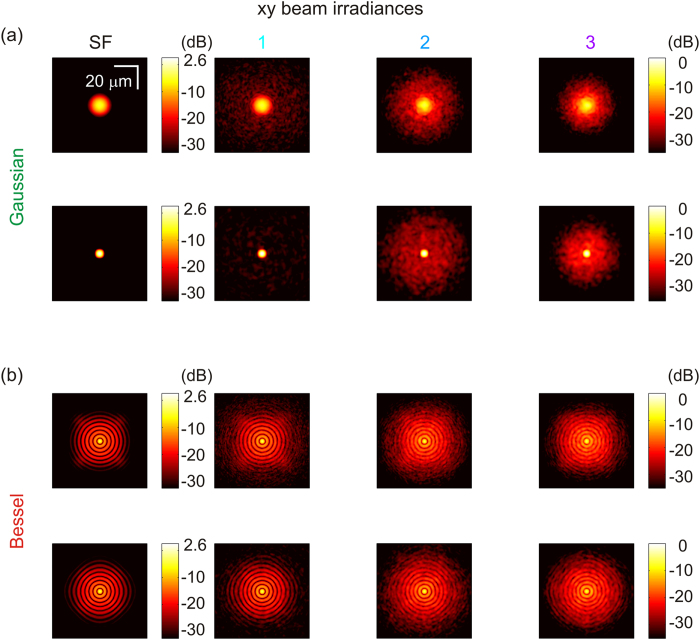
Simulated transverse beam profiles. Degradation of an ensemble-average of 10 transverse beam profiles for (**a**) Gaussian, and (**b**) Bessel beams on a logarithmic irradiance scale, where 0 dB represents the peak Gaussian irradiance (at focus) attenuated by the overlayers. Transverse profiles are plotted for two depths, shown in Fig. 2, corresponding to 60 μm before the focus, and at the focus (top and bottom, respectively.)

Figure 3 shows an ensemble-average of 10 transverse beam irradiance profiles plotted at two different depths (60 μm before the focus and at the focus.) Remarkably, the lateral extent and magnitude of the background are similar for both beams. Also, the side-lobe structure of the Bessel beam becomes increasingly buried in the background at high anisotropies (Cases 2 and 3). We found qualitative agreement between the experimental and simulated transverse PSFs (plotted on a logarithmic scale in Supplementary Fig. S7), compatible with the necessarily different realizations of the coherent (speckled) field between the two. We subsequently evaluated the SBR on the simulated beam irradiances of Fig. 3.

**Figure 4:**
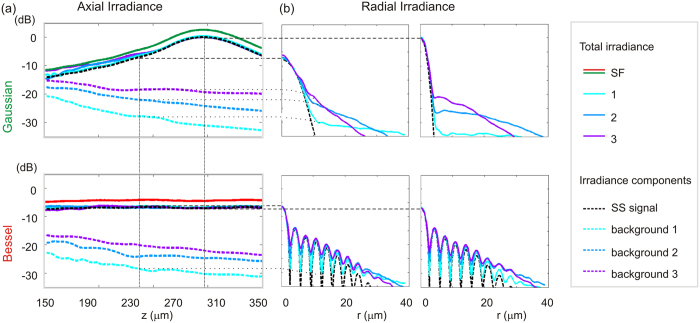
Simulated total beam irradiance and image-carrying and -degrading components of the beam versus depth in the PSF phantom after each overlayer. (**a**) Axial ensemble-averaged profiles, for the (top) Gaussian and (bottom) Bessel beams on a logarithmic irradiance scale. (**b**) Radial averages of the ensemble-averaged transverse Gaussian and Bessel beam profiles on the same scale for the two depths indicated by the dashed lines in Fig. 2.

Figure 4 shows the computed beam axial and radial logarithmic irradiance plots, in which the solid lines represent the total irradiance immediately after propagation through each overlayer in the PSF phantom. The black dashed line represents the image-carrying (or single-scattering, SS) component of the total irradiance and, by design, it does not vary with the scattering layer. The on-axis image-degrading component (or background) is shown for both beams after each overlayer using colour dashed lines in the axial plots in Fig. 4(a). To estimate the axial background, we used the radial plots in Fig. 4(b). The radial plots are averages over radial lines for 500 polar angles for each of the transverse plots of Fig. 3(a,b). For the radial plots, for both the Gaussian and Bessel beams, the coherent background is directly identifiable off-axis and can be extrapolated with a quadratic fit to estimate its on-axis contribution. This is even more conveniently done for the Bessel beam, as we can evaluate the background alone as close as 2.7 μm from the optical axis, where the ideally attenuated diffraction-limited beam irradiance is close to zero. Therefore, we calculated the average axial background irradiance at each depth over the same 200 μm range of propagation in the PSF phantom after the overlayer. The results in Fig. 4 demonstrate that both beams have similar on-axis image-degrading components, of increasing magnitude with increasing anisotropy. As the PSF phantom bulk provides no attenuation, the contribution of both beams decreases with depth in the PSF phantom due to divergence of the forward-scattered light.

With both the image-carrying and image-degrading axial components, we can plot the on-axis SBR versus depth, *z*, as:


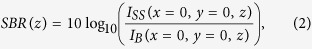


where the image-carrying component for either beam after any overlayer is *I*_*ss*_(*x, y* = 0, *z*) = *I*_*sf*_*(x, y* = 0, *z*)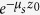, i.e., the scattering-free irradiance ideally attenuated by 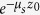.

**Figure 5:**
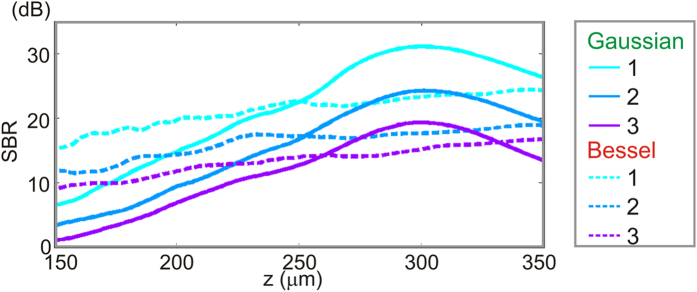
On-axis signal-to-background ratio (SBR) versus depth for Overlayers 1**–**3.

Figure 5 shows the on-axis SBR in the PSF phantom for both beams after propagation through each overlayer. An important result confirmed by Fig. 5 is that a higher anisotropy implies a smaller SBR regardless of the beam type. The smaller the SBR, the smaller the ratio of the image-carrying to image-degrading component of the detected backscattered light. This means that, in a scattering sample (such as the contrast phantom), any sample structure (refractive index variation) illuminated by the much larger background irradiance will increasingly contribute to the image, even if it falls outside the diffraction-limited spot, therefore, reducing the OCT contrast.

Another result taken from Fig. 5 is that, for any given value of *g*, we observe a higher susceptibility of Bessel beams to turbid tissue scattering, i.e., a lower SBR than that of the Gaussian beam in focus. This is due to the reduced on-axis irradiance of a Bessel beam (see Supplementary Fig. S1), which reduces only the image-carrying component, i.e., the numerator of the SBR. This susceptibility is mitigated in regions outside the Gaussian focus, where these power ratio considerations, rather than the self-reconstructing property, enable the Bessel beam to regain a higher SBR than the Gaussian beam.

### OCT contrast degradation

**Figure 6:**
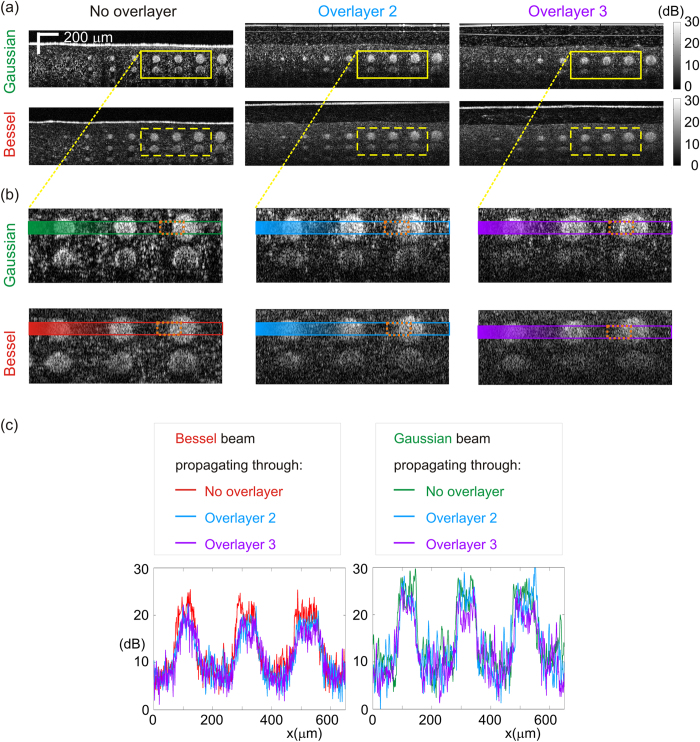
OCT contrast assessment. (**a**) Experimental OCT B-scans of the contrast phantom acquired with Gaussian and Bessel beams propagating through the overlayers (Overlayer 1 not shown). (**b**) Close-up (650 × 250 μm) on selected pillar features, boxed by a yellow line in (**a**). (**c**) Transverse line across selected pillars in the beam focal region. Lines are averages over depth within the areas bounded by the respective coloured boxes. Quantitative contrast in Table 1 is evaluated using the 80 μm × 40 μm orange box around the pillar edge.

Figure 6(a) shows experimental OCT images, for both beams, of the contrast phantom for the case with no overlayer (left) and two cases with scattering overlayers. For the case with no overlayer, a neutral density filter with optical density 0.25 was used to attenuate the beam to obtain an SNR in the contrast phantom comparable to that obtained with the scattering overlayers. Figure 6(b) shows close-up regions including the 60, 70, and 80 μm-wide pillars, boxed by a yellow line in Fig. 6(a), for the scattering-free case, and for Overlayers 2 (g = 0.987) and 3 (g = 0.993) (Overlayer 1 omitted for clarity).

Figure 6(c) shows transverse lines centred on the first row of pillars, for the three selected pillars, in an area close to the beam focus. The lines are incoherently averaged over 40 μm-long segments of A-scans. To aid the visual comparison of contrast degradation on a logarithmic scale, for each curve, the mean signal from the embedding casting is normalized to that of the corresponding beam for Overlayer 2. We performed the contrast assessment by analysing the pillar-to-embedding casting contrast, *C,* in an area boxed by an orange dashed line, ~80 μm × 40 μm across the left edge of the largest pillar. The maximum contrast, *C*_*0*_, was attained by the Gaussian beam without overlayer with a value of *C*_*0*_ = 12.6 dB. The reduction in contrast calculated as the ratio of the contrast *C* to the best case *C*_*0*_, i.e., *C/C*_*0*_, expressed on a linear scale, is presented in Table 1.

The results quantify the contrast degradation that takes place with increasing anisotropy for both beam types, which reaches, at its worst, ~60% of the contrast at focus (for a given beam) in the case with no overlayer, for the given parameters.

The results highlight that, around the focus, the degradation is lower for the case of the Gaussian beam. This is explained by the higher SBR for the Gaussian beam at focus, which translates to a contrast advantage. In fact, at that depth, both beam types produce a similar image-degrading background, but the relative contribution of this background to the image formation process is smaller for the Gaussian beam at focus, owing to its peak irradiance advantage over the equivalent Bessel beam, making the Gaussian beam produce up to ~40% better contrast than the Bessel beam.

## Discussion

In this paper, we have brought a comprehensive suite of tools to bear on the question of the relative performance of Bessel and Gaussian beams in OCT imaging in turbid tissue-like media. Through the combination of a novel, rigorous full-wave electromagnetic simulation, a well calibrated dual-beam OCT setup, and a set of controlled scattering overlayers and a highly evolved image target phantom, we have calculated an important indicative parameter of contrast degradation, the on-axis signal-to-background ratio, SBR, and verified that lower SBR leads to higher contrast reduction in experimental OCT images.

The narrow range of high anisotropies (*g* = 0.94–0.99) tested here is in line with previous analytical, simulated and experimental studies in OCT of the image-degrading background due to forward scattered light^11^,^34^, using micron-sized polystyrene particles. In our case, the lower refractive-index contrast (Δ*n* = 0.06) and the mixture of particle diameters used (*d* = 1.3–5.5 μm) more closely approximate cell nuclei and other sub-cellular and cellular tissue constituents that dominate the scattering properties of tissue^22^.

The reduction of contrast with increasing anisotropy was postulated by Schmitt *et al*., considering only the Gaussian beam case^5^. We evaluated the SBR similarly to the ratio of single back-scattered-to-multiple back-scattered amplitude, 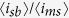, in equation 29a) of Schmitt *et al*.^5^, which gives a quantitative predictor of contrast reduction. We consider the same scattering coefficient and thickness for all overlayers (same scattering parameter). Therefore, in our case, that ratio would reduce to 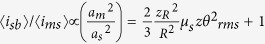, where *a*_*m*_ is the width of the overall PSF, *a*_*s*_ is the width of the diffraction-limited PSF, *z*_*R*_ is the Rayleigh range of the focused beam, *R* is the 1/e radius of the collimated beam impinging on the objective lens and *θ*_*rms*_ is the root-mean-squared scattering angle. This is the ratio of the width of the total PSF to the width of the diffraction-limited PSF (image-carrying), i.e., it scales with the width of the multiply forward-scattered PSF (image-degrading). Due to the energy conservation of the elastic scattering process, a smaller background width leads to a larger on-axis background peak irradiance, which is the parameter we chose to evaluate in the denominator of the SBR. For lower root-mean-squared scattering angles *θ*_*rms*_ (higher *g*), the background width is smaller and so is the ratio 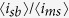, or, equivalently, the on-axis background irradiance is higher and so the SBR is reduced. This means that our choice of using the on-axis ratio of image carrying-to-image degrading irradiances for the SBR is consistent with the methods and findings of previous research in the field, but it also enables comparison of SBR with beams that do not have a transverse Gaussian profile.

To answer the question of what beam type attains better contrast in turbid tissue (higher SBR), in the situation where there is no *a priori* knowledge of the depth-dependent phase and amplitude spatial response of the sample refractive index distribution, and no adaptive beam correction, we have made a few assumptions that we recap here.

We considered Bessel and Gaussian beams of equal power and FWHM width and assumed that their focus (or, more accurately, their DOF centre) is placed at the same depth in the sample. Thus, they experience the same peak attenuation, determined by the scattering parameter (at the given NA). Indeed, we demonstrated that the overall scattered power is then very similar for both beams, as visible in Fig. 4(a). Also, we showed that the spatial distribution and concentration around the optical axis of the forward-scattered component is very similar for both beams and determined by the scattering anisotropy.

**Figure 7:**
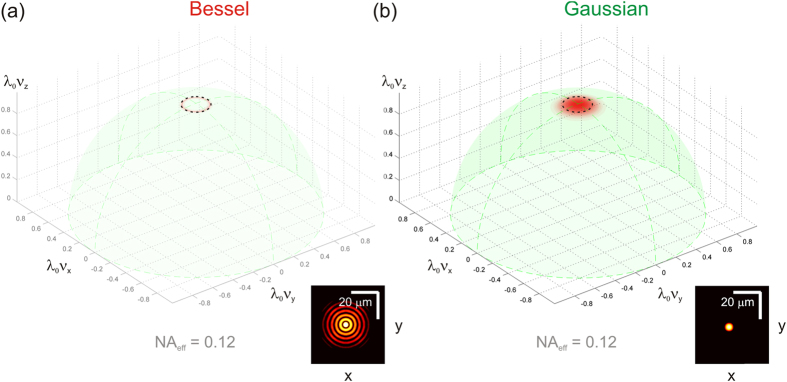
Angular spectra intensity of the beams. (**a**) Bessel beam. The small inset shows the PSF at DOF centre. (**b**) Gaussian beam. The small inset shows the PSF at focus. Both beams have an effective NA_eff_ = 0.12, indicated by the locus of wavevectors corresponding to 1/e^2^ spatial frequency spectral width.

The components of the Bessel beam’s angular spectrum propagate within a narrow range of angles with respect to the optical axis. This fact has two important ramifications: the Bessel beam’s extended DOF and its ability to self-reconstruct. Firstly, all components maintain a similar phase as they propagate along the optical axis, thus, resulting in an extended DOF. An illustration of the angular spectra of the Bessel and Gaussian beams, and the corresponding PSF, is shown in Fig. 7. We hypothesize that Bessel beams do not reconstruct their profile any better than Gaussian beams in turbid media that exhibit distributed scattering that approximates that of biological tissue. This is because in tissue-like turbid media, all sections of each component of the Bessel beam angular spectrum will be perturbed and so perturbed wavefronts will reach all axial locations, thus, preventing self-reconstruction. This situation differs markedly from that in which an isolated point scatterer is encountered, when the self-reconstructing property is in evidence^17^.

This hypothesis is supported by the fact that both beams produce a nearly identical background, at least for the case in which the obstructions (PMMA spheres in present case) are distributed throughout an area covering most (>95% here) of the beam input energy. These disrupting backgrounds increase in scale with anisotropy, as visible in Fig. 2.

Yet Bessel beams have been shown to perform well in turbid tissue^14^. We believe that the reported persistence of the central lobe, i.e., its propagation stability^33^, is not a result of the self-reconstructing property of the Bessel beam but rather a consequence of the ratio of the power confined within the central lobe (in the absence of scattering) to the scattered power being substantially high. More specifically, the power fraction confined within the central lobe is determined by the Fresnel number^16^, and the scattered power is determined by the scattering parameter (at the given NA). The combination of low Fresnel number (resulting in a high proportion of the beam power being confined in the central lobe) and low scattering parameter is all that is necessary to explain the central lobe propagation stability in distributed scattering media.

Another consequence of this analysis is that the lower peak irradiance of the Bessel beam (see Supplementary Fig. S1), relative to a Gaussian beam of the same power, is then buried in a similar image-degrading background, leading to lower on-axis SBR and lower OCT image contrast around focus than those produced by the Gaussian beam.

The Bessel beam also suffers an additional contrast penalty at focus, even in the scattering-free case, as the side-lobe structure of Bessel beams inherently reduces contrast compared to the equivalent Gaussian beam (see Fig. 6 and Table 1). Indeed, the combination of Bessel beam illumination and Gaussian beam detection has been used, in part, to address this 14. However, below the focal region of the Gaussian beam, the Bessel beam holds an irradiance advantage (see Supplementary Fig. S1) and, consequently, an SBR advantage (far right side of Fig. 5), which ultimately translates to a contrast advantage. This can be seen by looking at the second row of pillars in Fig. 6(b), which shows better delineation of the pillars in images formed by the Bessel beam, both in terms of resolution and contrast. (Contrast quantification in this area is not reliable because of the low SNR at this depth.)

We can readily extend our results on SBR and its relation to contrast degradation when the condition that both beams have the same input power is relaxed. Allowing an increase in relative power of the Bessel beam only affects the relative OCT SNR, but not the contrast between features in the image, as doubling the Bessel beam power, for example, will also double the coherent background produced by the overlayer, and their ratio will be independent of the input power.

One significant advantage of Fourier-domain OCT over other microscopy techniques is the high 3D acquisition speed afforded by the absence of mechanical movement of the sample arm optics (or sample) required to acquire a depth profile. A great advantage of a Bessel beam is its longer DOF. For these reasons, the use of a Bessel beam may still be advisable, as the contrast reduction around the DOF might be an acceptable trade-off for high-speed acquisition, depending on the OCT system sensitivity. At the same time, our results suggest that, if acquisition speed is not critical, and combatting image-degrading tissue scattering is a priority, dynamically focusing a Gaussian beam is preferable to use of a Bessel beam in retaining higher OCT contrast at depth (and the same resolution).

Dynamic focusing removes the condition that the centre of the DOF be aligned at a certain depth inside the sample. From simple geometrical considerations, if the Gaussian beam focus is placed deeper in the sample, the beam will encounter more scatterers at any given depth, due to its larger beam cross-section than in the shallower focus case. Hence, as the Gaussian focus is placed deeper in the sample, the effective scattering , i.e. the equivalent scattering parameter for a collimated beam geometry, increases. With an increasingly higher effective scattering parameter, more and more light is scattered and the background increases, leading to a decrease in the SBR, until the Gaussian beam SBR at focus reduces to that of the Bessel beam with its focus in the original position. Below this depth, there will be no contrast advantage of the Gaussian focus over the Bessel beam. The effect of increasing effective scattering parameter with depth would be more prominent in high-numerical aperture regimes, for a given sample^35^, reducing the range over which shifting the Gaussian focus deeper produces any contrast benefit relative to an equal-NA Bessel beam. However, our results suggest that, for sufficiently low numerical aperture (NA < 0.1) and typical soft tissue properties (*μ*_s_ = 3.7 mm^−1^ and high scattering anisotropy, *g* > 0.95), superior contrast (by up to ~40%) may be obtained by a Gaussian beam combined with dynamic focusing over an extended depth range. The depth at which the contrast crossover occurs within representative soft tissues, as a function of the NA, will be the subject of further studies.

In conclusion, we analysed the effect of using Bessel or Gaussian beams on OCT contrast in turbid tissue for a range of scattering anisotropies. We rigorously compared the performance of both beam types using beams of equal resolution, input power, and aligned foci in the sample. We did this by analysing both simulated beam propagation and experimental images of scattering imaging targets through overlayers that produce the strong forward scattering typical of biological tissue.

We demonstrated that, the higher the scattering anisotropy of the overlayers, the lower the OCT contrast, regardless of the beam type. This effect is marked by a reduction of the SBR, the on-axis signal to background ratio, which is, in effect, the ratio of the image-carrying to image-degrading signal components. The forward-scattered background has a higher on-axis irradiance with increasing *g*, as its divergence ‘cone’ is increasingly concentrated around the optical axis.

Furthermore, the Gaussian beam in focus suffers less reduction of local contrast than does the Bessel beam. For equal powers, this is caused by the inherent lower on-axis peak irradiance of a Bessel beam necessary for it to have an extended depth of field. On the other hand, the irradiance of the forward-scattered background of both beams is very similar, which means that the Bessel beam is just as sensitive to scattering as the Gaussian beam, and it does not reconstruct any better than a Gaussian beam in turbid tissue. This implies that the SBR for a Bessel beam anywhere along its optical axis is bound to be lower than the SBR for a Gaussian beam in focus, when their foci are at the same depth in the sample. We conclude that, in cases where Bessel beams have been reported to exhibit propagation stability in turbid tissue, this characteristic is more related to a relatively large fraction of the beam power being confined in the central lobe (achieved by using a low Fresnel number), than to the self-reconstructing property.

## Methods

### Beam and Sample Configuration

#### Optical beam characteristics

We computationally and experimentally realized Gaussian and Bessel beams of equal centre wavelength (840 nm), numerical aperture (0.12, FWHM resolution 2.6 μm) and power, but with different DOF, and peak irradiance (see Table 2, and Supplementary Fig. S1.) The centre of the DOF of both (the focus in the case of the Gaussian beam) was located at the same depth in the sample, so that both beams experienced the same effective scattering parameter, *μ*_*s*_*z*_*0*_ 12.

#### Scattering overlayers

We manufactured and simulated three overlayers as sources of controlled, turbid tissue-like scattering, following the method of Bisaillon *et al*.^25^. The overlayers are 150 μm-thick silicone slabs containing a uniform-density, random distribution of PMMA spheres. The spheres in each overlayer have different nominal diameters and concentrations, chosen to produce the same scattering coefficient, *μ*_*s*_ = 3.7 mm^−1^ and different anisotropy *g* (see Table 3, and Supplementary Fig. S2), as calculated from Mie theory. The refractive indices, sphere sizes, resulting scattering coefficient, and thickness were chosen to be representative of typical human soft tissue, such as skin, muscle or epithelial tissue^21^,^36^. To simulate each overlayer, ten different random sphere arrangements were computationally generated, enabling an ensemble-averaged measure of the irradiance of the beam after propagation through the overlayer.

#### Imaging targets (phantoms)

To test the imaging PSF, we used a target (phantom) consisting of randomly dispersed 300 nm–800 nm-diameter, red iron-oxide (Fe_2_O_3_) particles (*n* ≈ 3) embedded in polyurethane resin (*n* = 1.49) (National Physical Laboratory, UK)^37^, shown in Supplementary Fig. S3.

To assess OCT contrast, we obtained a purpose-manufactured imaging target (contrast phantom) with well-specified geometry based on silicone mixed with TiO_2_ particles. The average particle size was 1 μm. Consecutive rows of pillars protrude into an embedding silicone casting, doped with a different concentration of TiO_2_ particles (see Supplementary Fig. S3). The pillars in each row vary from 10 to 90 μm in diameter and are spaced at a pitch of 200 μm. Each row is spaced at a pitch in depth of 100 μm. The manufacturing technique was replica-molding soft lithography^38^.

With regards to equation (1), we expect a nominal maximum OCT contrast C = 10 dB for a 10-fold concentration difference between the pillar and the embedding casting, as the average OCT signal amplitude scales nominally with the square root of the scatterer concentration^25^,^39^, i.e. 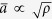 , (Table 4).

### Analysis Implementation

#### Simulation

The pseudo-spectral time-domain (PSTD) method^40^,^41^ was used to perform the EM simulations of the two beam types within the overlayers. We then propagated the output field for a further 200 μm using an angular spectrum method^42^, as no significant bulk scattering is expected in the PSF phantom (see Supplementary Table S1, and Supplementary Fig. S4). Our computational analysis of the SBR was limited to the illumination irradiances, they were adequately representing the trend of PSF degradation with increasing anisotropy whilst the complexity of the simulations was still contained.

#### Experimental

In the experiment, we used a spectral-domain OCT system (see Supplementary Fig. S5) with a superluminescent diode light source (Superlum), centred at 840 nm wavelength with a 3 dB bandwidth of 50 nm. It featured a reconfigurable sample arm to produce both beam types.

### Validation

The procedure and results of the validation are detailed in the Supplementary Information online. We confirmed that both the simulated and experimental beams and overlayers are in agreement with our specifications, with minor exceptions. In particular, the single scattering predominance in the imaging targets was confirmed (see Supplementary Fig. S6).

## Additional Information

**How to cite this article**: Curatolo, A. *et al*. Quantifying the influence of Bessel beams on image quality in optical coherence tomography. *Sci. Rep.* **6**, 23483; doi: 10.1038/srep23483 (2016).

## Supplementary Material

Supplementary Information

## Figures and Tables

**Figure 1:**
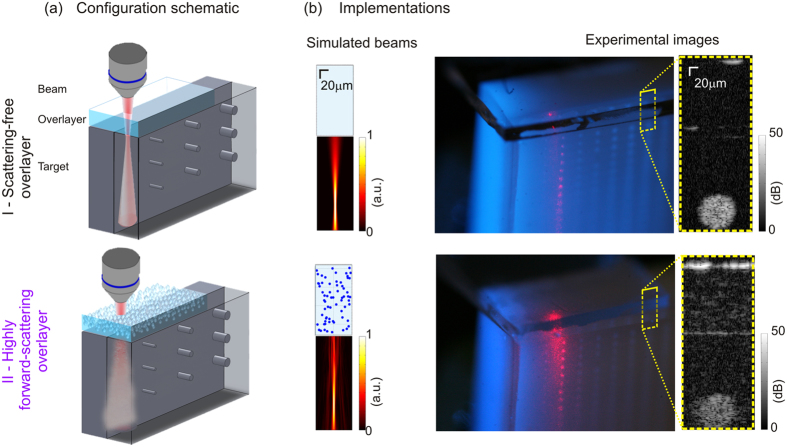
Beam and sample configuration. (**a**) Schematic diagram of the beam, overlayer and imaging target, for transparent (top) and scattering overlayers (bottom). (Not to scale and not all pillars shown.) (**b**) The configurations implemented computationally (left), by simulating the one-way beam propagation through the overlayers, and experimentally (far right), by acquiring OCT images, here shown from an area bounded by a yellow dotted box in a macro-photograph (middle) of the target illuminated with a red beam.

**Table 1:** Contrast reduction for each beam type and overlayer.

	**Gaussian**	**Bessel**
No overlayer	1	0.87
Overlayer 2	0.79	0.65
Overlayer 3	0.72	0.51

**Table 2:** Simulated and experimental beam characteristics.

**Beam type (in free space)**	**Gaussian**	**Bessel**
Transverse resolution, FWHM	2.6	2.6
Depth of field (μm)	40	330
Irradiance penalty (dB)	0	7.5

**Table 3:** Specified and calculated scattering overlayer characteristics.

**Overlayer**	**1**	**2**	**3**
Medium refractive index	1.42	1.42	1.42
Sphere refractive index	1.48	1.48	1.48
Sphere diameter (μm)	1.3	3.36	5.5
Sphere concentration (x10^3^ μm^−3^)	16,700	400	69
Scattering coefficient *μ*_*s*_ (mm^−1^)	3.7	3.7	3.7
Scattering anisotropy *g*	0.946	0.987	0.993

**Table 4:** Contrast phantom nominal characteristics.

	Casting withpillars	**Embedding casting**
Medium refractive index	1.42	1.42
Scatterer (TiO_2_) refractive index	2.51	2.51
Scatterer concentration ρ - (mg/ml)	5	0.5
Scattering anisotropy *g*	0.36	0.36
OCT nominal contrast *C* (dB)	10	
