# Supplementary material for: Quantifying the influence of Bessel beams on image quality in optical coherence tomography
Source: Sci Rep. 2016 Mar 24;6:23483. doi: 10.1038/srep23483 (PMC4806300; doi:10.1038/srep23483)
Supplement: Supplementary Information [file srep23483-s1.pdf]

Supplementary information

Quantifying the influence of Bessel beams on image quality in optical coherence tomography

Andrea Curatolo[1,*], Peter R. T. Munro[1,2,3], Dirk Lorenser[1], Parvathy Sreekumar[1], C. Christian Singe[1], Brendan F. Kennedy[1] and David D. Sampson[1,2]

[1]Optical+Biomedical Engineering Laboratory, School of Electrical, Electronic & Computer Engineering, The University of Western Australia, 35 Stirling Highway, Crawley WA 6009, Australia

[2]Centre for Microscopy, Characterisation & Analysis, The University of Western Australia, 35 Stirling Highway, Crawley, WA 6009, Australia

[3]Current Address: University College London, London WC1E 6BT, United Kingdom

*Correspondence:

Mr. Andrea Curatolo,

Optical+Biomedical Engineering Laboratory,

School of Electrical, Electronic & Computer Engineering,

The University of Western Australia, M018, 35 Stirling Highway, Crawley WA 6009, Australia.

Tel: (+61) 08 6488 3916,

Email: andrea.curatolo@uwa.edu.au

Methods

Beam and Sample Configuration

Optical beam characteristics

We realized Gaussian and Bessel beams with identical effective numerical aperture of 0.12 (FWHM resolution of 2.6 μm) but with different depth of field (DOF). The beams have equal centre wavelength, power, and their respective DOF centres, i.e., the midpoint between the locations of the axial irradiance half-maxima, are aligned in the sample. The DOF centre for the Gaussian beam corresponds to the focus position, whereas, for the Bessel beam, it is located after its peak axial irradiance, due to its asymmetrical profile.

The Gaussian and Bessel beams were simulated (in free-space) using both commercial optical modelling software (VirtualLab, LightTrans International UG), and a custom-built numerical solver of Maxwell's equations of electromagnetism (EM)[1]. Experimentally, the beams were produced using reconfigurable optics in an OCT imaging system set up in reflection-mode, i.e., the beams used the same path for illumination and collection. The beams were measured using a commercial beam profiler (SP620U, Ophir-Spiricon, USA).

Supplementary Fig. 1 shows the simulated beam profiles and the beam axial and radial irradiance plots, for both simulation and experiment. Supplementary Fig. 1(a) shows the magnitude of the electric field component along the x-axis, $|E_x|$, across a plane transverse to the optical axis (aligned with the z-axis) for both beams.

This represents both an output of the VirtualLab simulation for the reconfigurable sample arm, and, together with the much weaker E_y component on the same plane (not shown), the input for the full EM numerical solution simulation. The output of the simulation is shown in Supplementary Fig. 1(b)-(c), with phase[2] and amplitude of the electric field E_x of both beams displayed in quarter-sections with the proximal edge coinciding with the optical axis.

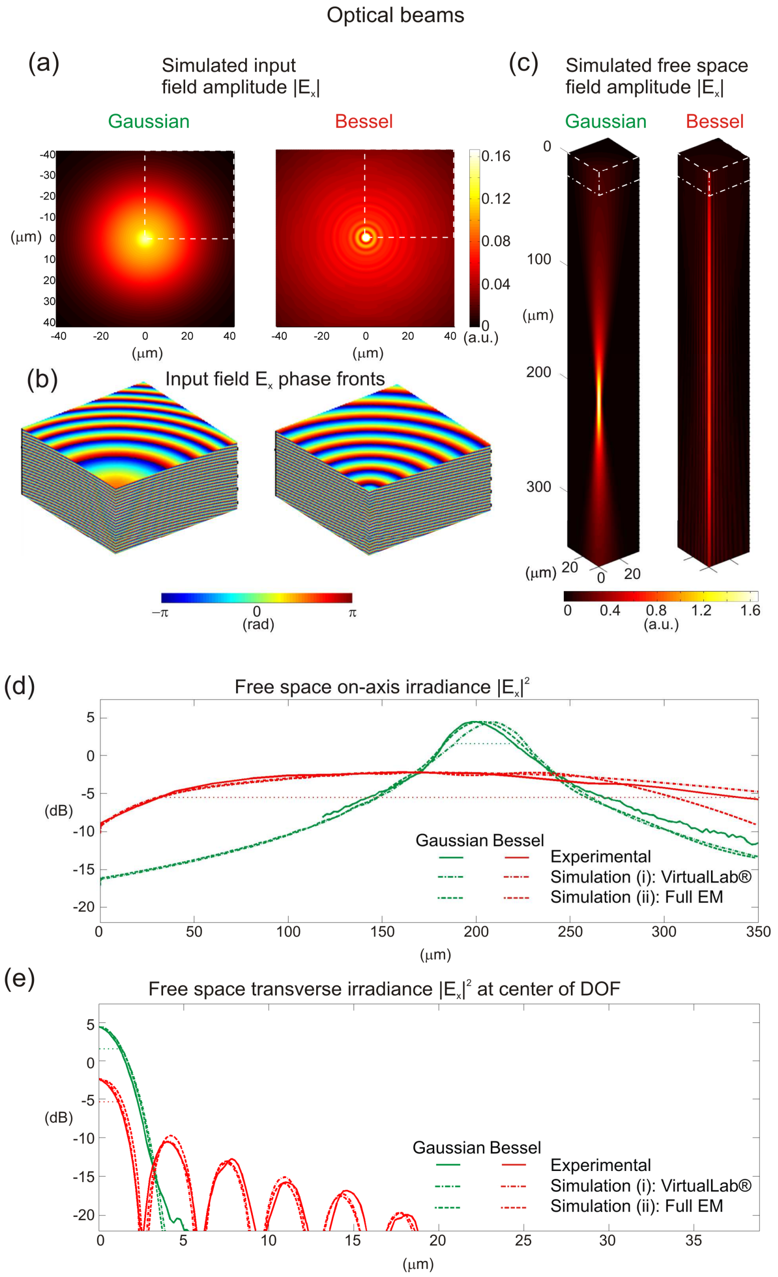

Supplementary Figure 1. Optical beams: Gaussian and Bessel beams of equal resolution and power. (a) Simulated input electric field $|E_x|$ amplitudes for the Gaussian and Bessel beams, and (b) typical quadratic and linear field phase profiles in the quarter sections bound by a white dashed line in (a). (c) Simulated free space electric field amplitude $|E_x|$ in a 3D quarter section of the full simulation workspace. The proximal edge at $(x,y) = 0$ coincides with the beam optical axis. Simulated and experimental free space (d) on-axis and (e) radial logarithmic irradiance, where 0dB represents the peak Gaussian irradiance attenuated by the overlayers. There is a good match between simulated and experimental beam profiles.

Scattering overlayers

We manufactured three overlayer tissue phantoms as sources of controlled turbid tissue-like scattering, following the method of Bisaillon *et al.*[3]. Each comprised a 150 μm-thick slab of room-temperature vulcanizing (RTV) silicone (RT601, Wacker, Germany, group refractive index n_{med} = 1.42), containing a mono-distribution of poly-methyl methacrylate (PMMA) spheres (Bangs Laboratories, USA, group refractive index n_{sp} = 1.48) with varying diameters and concentrations to produce the same scattering coefficient μ_s = 3.7 mm^{-1}, calculated from Mie theory, but differing anisotropy. Additionally, we manufactured a transparent, scattering-free silicone overlayer for comparison, to compensate for the axial focal shift and spherical aberration taking place in the silicone matrix of the scattering overlayers. Attributes of the simulated and experimentally realized overlayers are shown in Supplementary Fig. 2.

For computational efficiency in the simulation, a volume section intersecting, in each case, at least 95% of the beam power was represented, resulting in a simulation volume of 80 x 80 x 150 μm (*x y z*). Ten different realizations of the sphere distribution in the overlayers were computationally generated for each overlayer type, to be able to obtain an ensemble-average measure of the irradiance of the beam after propagation through the overlayer. One of those realizations is shown for each of the four overlayers in Supplementary Fig. 2(b). Supplementary Fig. 2(c) shows the logarithmic Mie theory phase function plots for the spheres employed in each of the three overlayers, normalized to the forward direction, providing a visual representation of the scattering anisotropies tested.

Imaging targets (phantoms)

Supplementary Fig. 3(a) shows a photograph of the point spread function (PSF) phantom illuminated by a Bessel beam. On the right hand side, a schematic provides a representation of the area density of randomly dispersed 300 - 800 nm-diameter, red iron oxide (Fe_2O_3) scatterers ($n \approx 3$) embedded in polyurethane (n = 1.49) resin (National Physical Laboratory, UK)[4].

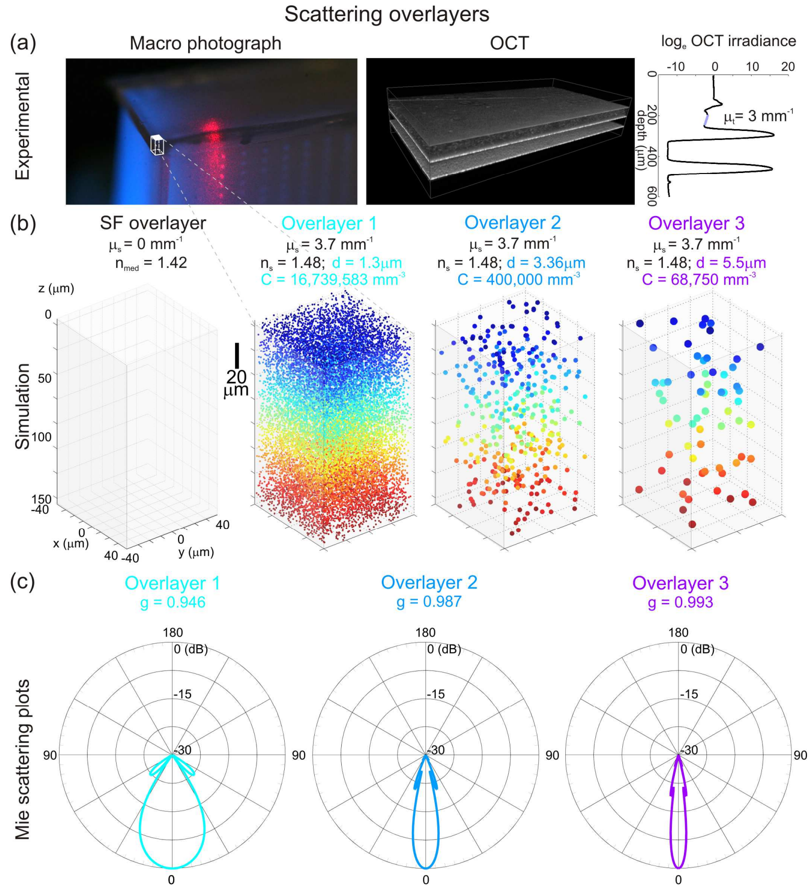

Supplementary Figure 2. Scattering overlayers. Three different mono-dispersions of PMMA spheres (n = 1.48) in RTV silicone (n = 1.42), providing the same scattering coefficient (μ_s = 3.7 mm^{-1}) but increasing scatterer size and, therefore, scattering anisotropy. The overlayers are nominally 150 μm thick. A sphere-free (SF) silicon overlayer (at left) is used for comparison. (a) Experimental and (b) simulated overlayers with equal sphere concentration. The transverse area (80^2 μm^2) of the volume intersects 95% of the input beam power. Colour codes for depth. (c) Mie theory phase function plots for the spheres employed in each of the scattering overlayers.

The contrast phantom, shown in Supplementary Fig. 3(b), is made of two polydimethylsiloxane (PDMS) silicone castings (n = 1.42), one embedded into the other. One casting features 18 rows of pillars, ranging in diameter from 10 to 90 μm, doped with 5 mg/ml concentration of 1 μm-diameter TiO_2 scatterers (n = 2.51), protruding for 50 μm (PL) into an embedding casting with 1/10 of the concentration of TiO_2 scatterers. This produces an OCT attenuation coefficient[3] μ_t of 6.4 mm^{-1} for the pillars and 0.64 mm^{-1} for the embedding casting.

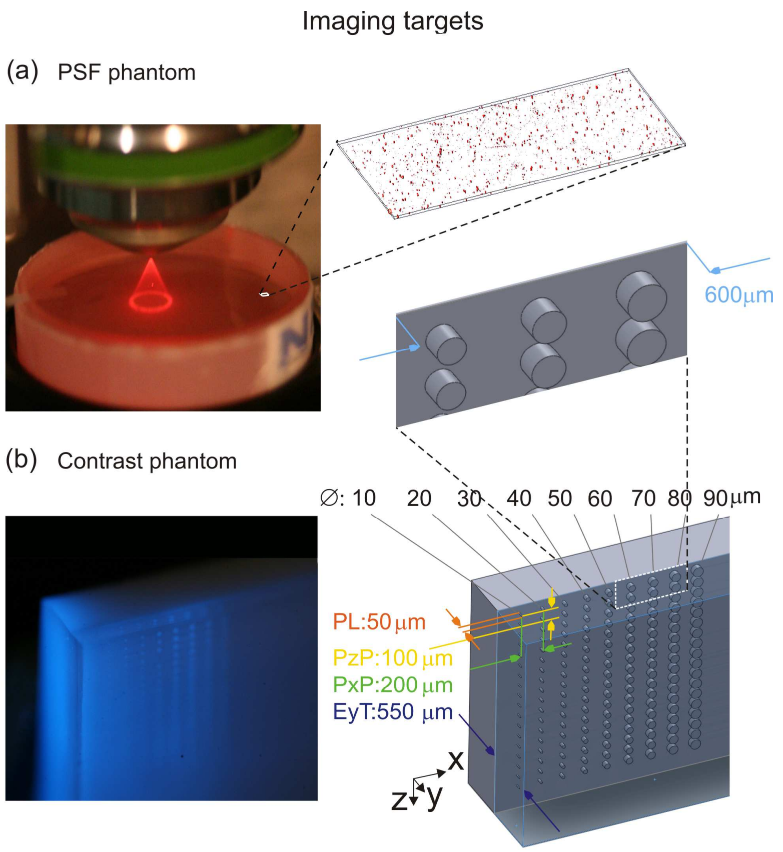

Supplementary Figure 3. Imaging targets. (a) Point spread function (PSF) phantom used for the analysis of PSF degradation with scattering. (b) Contrast phantom used for analysis of OCT contrast. On the left side of (a) and (b) are photographs of the phantoms. On the right side of (a) and (b), schematics of the two phantoms are shown. PL: protrusion length, PzP: pillar pitch along z, PxP: pillar pitch along x, EyT: embedding casting thickness along y.

The rows are separated by 100 μm (PzP), and each pillar is separated laterally by 200 μm (PxP). The embedding casting has optical quality surfaces with maximum distance of 550 μm (EyT) from the first row of pillars in all directions.

Analysis Implementation

Simulation

The pseudo-spectral time-domain (PSTD) method[5] was used to perform the EM simulations. The PSTD method uses the fast Fourier transform (FFT) to calculate spatial derivatives, instead of finite-differences used in the conventional finite-difference time-domain (FDTD) method[6], allowing significantly larger computational volumes to be simulated[7] with a Yee cell spatial grid spacing

approaching λ/2. For the sake of simplicity, we only used the PSTD simulation to calculate the field distribution inside the overlayers, and then propagated the output field for 200 μm into the PSF phantom bulk (polyurethane resin) using an angular spectrum propagation (ASP) method[8], as no significant bulk scattering is expected in the PSF phantom.

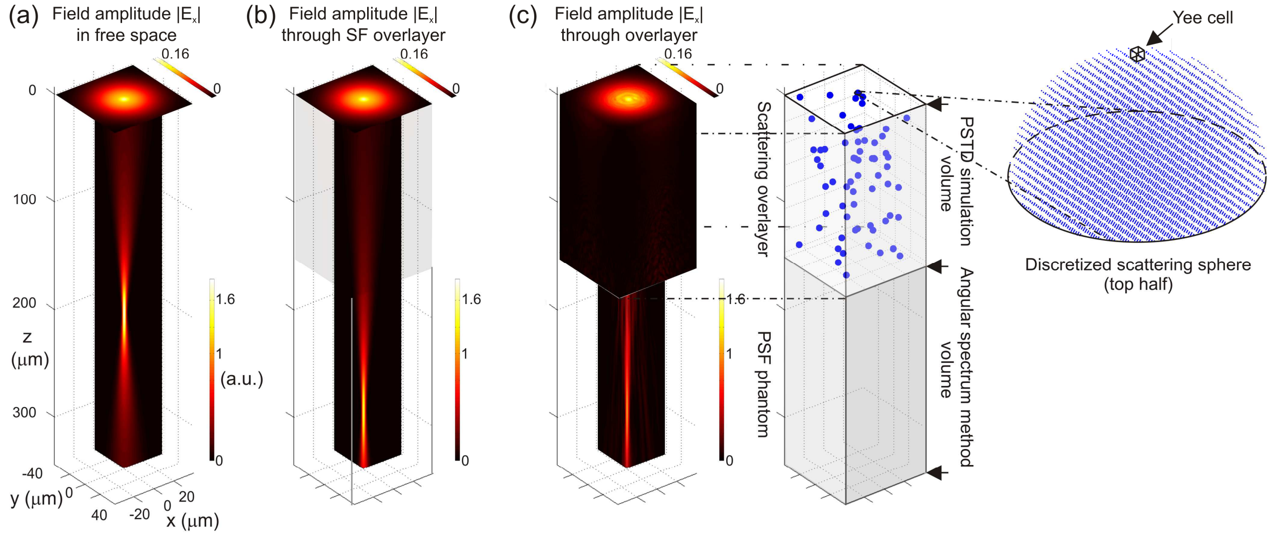

Supplementary Figure 4. Illustration of the EM simulation. The plots show the beam full transverse input field amplitude and its propagation over 350 μm in quarter section sliced along the optical axis. Gaussian beam propagation (a) in free space, (b) through scattering-free overlayer into the PSF phantom, and (c) through Overlayer 3 into the PSF phantom. Beam propagation in the overlayer is achieved with the PSTD simulation with a grid discretization (Yee cell) scale visible on the right. Beam propagation in the PSF phantom is achieved with an angular spectrum propagation method.

Supplementary Fig. 4 illustrates the simulation, by showing the Gaussian beam field amplitude in the input transverse plane and along a quarter section with the proximal edge coinciding with the optical axis. The beam propagation is shown in free space in Supplementary Fig. 4(a), through the SF overlayer (represented by a shaded gray box) into the PSF phantom in Supplementary Fig. 4(b), and through Overlayer 3 into the PSF phantom in Supplementary Fig. 4(c). In Supplementary Fig. 4(c) the faint scattered field amplitude on the boundaries of the PSTD simulation volume is also plotted. Supplementary Table 1 summarizes the simulation specification.

Yee cell size (nm)	210 (λ/4)
# of simulation cells	10^8
PSTD simulation volume x-y-z (μm)	80x80x150
ASP simulation volume x-y-z (μm)	80x80x200

Supplementary Table 1. Simulation characteristics

Experiment

In the experiment, we used a Fourier-domain OCT system, modified from that employed in Lorenser *et al.*[9], with a SLD light source (Superlum) centred at 840 nm wavelength with a 3 dB bandwidth of 50 nm. This bandwidth, assuming a Gaussian spectrum, results in a theoretical full-width at half-maximum (FWHM) axial resolution of 6.2 μm in air. The system's depth range was 3.11 mm in air. Using a fibre coupler, the source light is split 50:50 between sample and reference arm. The sample arm is reconfigurable, and incorporates a liquid crystal spatial light modulator (SLM, Pluto NIR II-HR, Holoeye Photonics).

The SLM was programmed, either with an axicon phase mask producing a low Fresnel number (N = 7.2) Bessel beam (effective focal length L_w = 106.5 mm) relayed to the sample via a 4f system (LB1 – LB2), or with a uniform phase mask conjugate, via another 4f system (LG1 – LG2), to the pupil plane of an objective lens (LG3), as seen in Supplementary Fig. 5. By appropriate combinations of input beam collimation, SLM phase mask and various focal lengths, we realized the Gaussian and Bessel beams previously described.

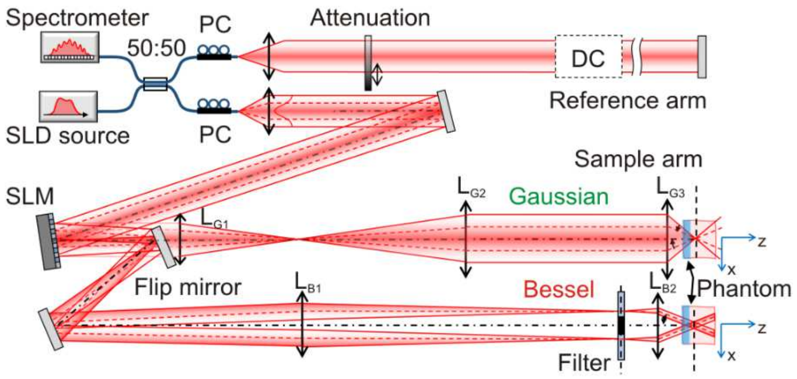

Supplementary Figure 5. Dual-beam OCT system schematic. PC = polarization controller, DC = dispersion compensation, SLD = superluminescent diode, SLM = spatial light modulator, LG1 = achromatic doublet lens (f = 100 mm), LG2 = achromatic doublet lens (f = 150 mm), LG3 = objective lens (f = 10 mm), LB1 = achromatic doublet lens (f = 300 mm), LB2 = achromatic doublet lens (f = 19 mm).

Validation

Optical beam types

The free-space axial and transverse irradiance plots of Gaussian (green) and Bessel (red) beams in Supplementary Fig. 1(d)-(e), respectively, show very good agreement between the VirtualLab and full EM simulations. This provided the first validation of the EM simulation for the beams in free-space. There is also good agreement with experimental curves, verifying the theoretical values given in Table 2 of the main manuscript. In these plots, 0 dB is the reference irradiance representing the Gaussian peak equally attenuated by all overlayers.

Scattering Overlayers

The scattering coefficient was initially estimated using Mie theory[10,11], from the input parameters given in Table 3 of the main manuscript, to be 3.7 mm^{-1}. It was verified computationally with a value $\mu_s \sim 4.0\ mm^{-1}$, by evaluating the ensemble-averaged Gaussian irradiance attenuation at focus, and solving equation (S1), the Beer-Lambert law, for the scattering coefficient, μ_s,:

$$I_{out} = I_{in} e^{-\mu_s z_0}, \quad \text{(S1)}$$

where I_{out} is the beam power after a single-pass through the overlayer, I_{in} is the beam power before the overlayer, and z_0 is the overlayer thickness. As we are dealing with a converging beam (not a collimated beam, as in the original formulation of equation (S1)[10]), comparing the irradiance on the optical axis before and after the overlayer would produce an erroneous result. A direct measurement of I_{in} would require a spatial integration and, for I_{out} , integration over a pinhole. For the sake of simplicity, a point measurement was chosen for the beam power evaluation. So, for consistency, we evaluated the focal on-axis irradiance after the scattering-free overlayer in place of I_{in} and the ensemble-averaged focal on-axis irradiance after the other overlayers for I_{out}.

The agreement between theory and computation was good and a scattering parameter, $d = \mu_s \cdot z_0$, close to 1 ensured that a parameter space in which single and multiple scattering coexist in the sample was being probed[12]. For Overlayer 2, we also experimentally measured the OCT attenuation coefficient, μ_t, to be $\mu_t \sim 3.0\ mm^{-1}$ (Supplementary Fig. 2(a)), obtained using the correction method described in

Scolaro *et al.*[13]. For Overlayer 3, we measured $\mu_t \sim 2.4$ mm^{-1}. The reason for the difference between μ_s (defined for a collimated beam) and μ_t measured by OCT and the decreasing trend of μ_t with increasing anisotropy is explained in Kalkman *et al.*[14]. Overlayer 1 had a slightly greater thickness, resulting in a higher effective scattering parameter, due to manufacturing tolerances.

By considering, for a given scattering coefficient, μ_s, the effect of increasing anisotropy on the measured OCT attenuation coefficient, μ_t, we could recognize the expected trend and confirm good agreement also between computational and experimental overlayers.

Imaging targets (phantoms)

To confirm the expected predominance of single scattering in the experimental imaging targets, we fit an analytical model to the experimental OCT signal intensity profile with depth. This procedure required different models for the two types of imaging target because the OCT system collects a higher average backscattered irradiance from a homogeneous scattering sample region (in the contrast phantom) than from isolated single scatterers (in the PSF phantom). In the former case, the resulting OCT signal effectively factors out the illumination confocal effect by integration of the concurrent responses of the many illuminated scatterers within the resolution volume[15,16]. In the PSF phantom, only a single (isolated) scatterer samples the illumination field over resolution-wide distances and the resulting OCT signal is influenced by the confocal effect both in the illumination and collection paths. This results in different depth profiles of the maximum and speckle-averaged OCT signal intensity for the PSF phantom and the contrast phantom, respectively.

Experimental OCT images of the PSF phantom are presented in Supplementary Fig. 6(a), for both beam types. The left hand panels of Supplementary Fig. 6(a) show close-ups of the scattering-free axial and transverse PSF near the focus. The location of the close-ups is highlighted in yellow in the co-registered B-scans in the middle panels. The right panels show a maximum intensity projection (MIP) of B-scans, along a 150 μm-thick portion of the *y*-axis of a C-scan. A MIP along the same portion of the *y*-axis and a 400 μm-thick portion of the *x*-axis (corresponding to the whole transverse B-scan range

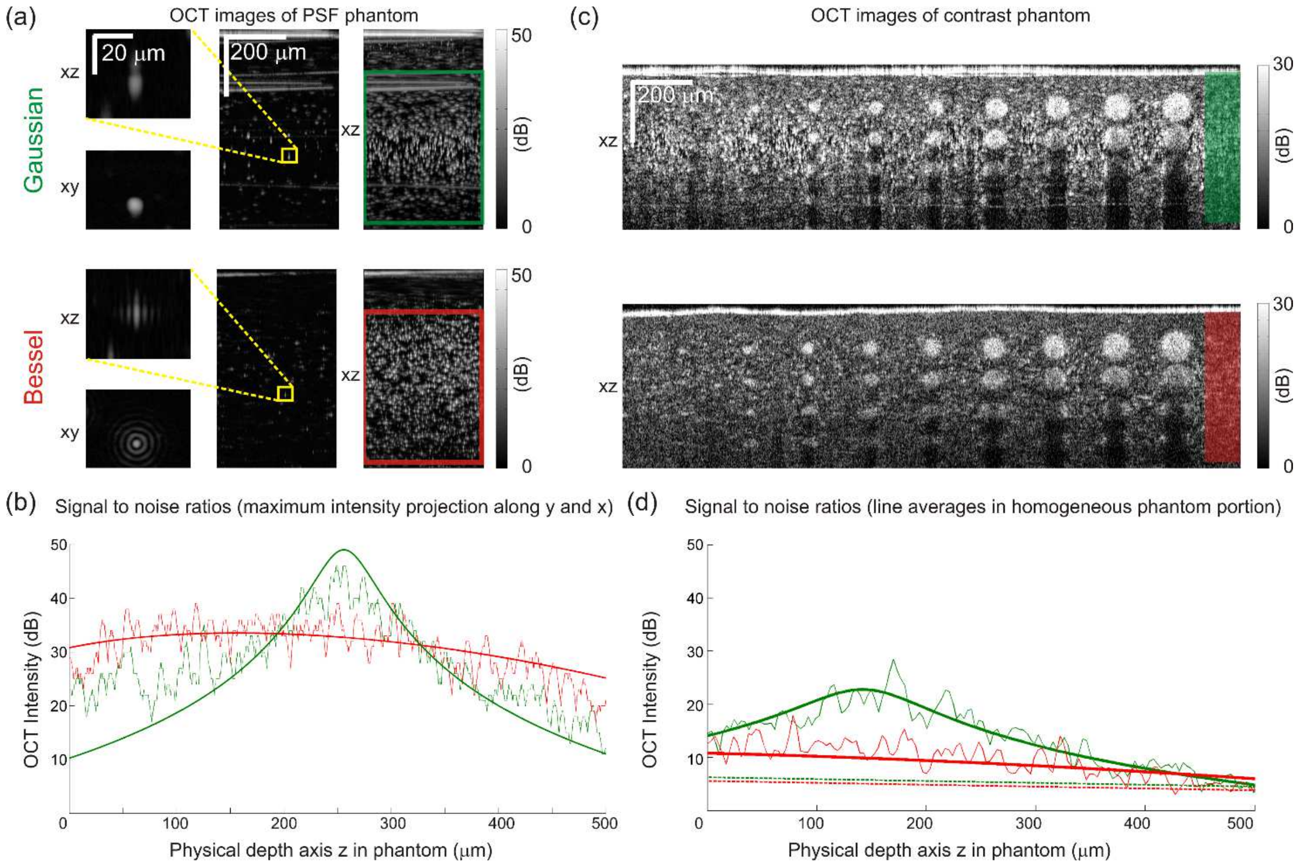

Supplementary Figure 6. OCT characterization of the imaging targets (no overlayers) with Gaussian and Bessel beams. (a) PSF phantom views from OCT C-scans. In the panels: (left) B-scan and *en face* images of a single PSF near the focus position; (middle) shown in a single co-registered B-scan; and (right) maximum intensity projection of B-scans along a 150 μm thick portion of the *y*-axis. (b) OCT peak axial intensity versus depth from an additional maximum intensity projection along a 400 μm thick portion of the *x*-axis (green and red boxed areas in (a)) with fits according to equation (S2). (c) Contrast phantom views from OCT B-scans. (d) OCT axial intensity laterally averaged over 100 μm (green and red highlighted areas in (c)) with fits according to equation (S3), where the dotted lines represent the limited OCT signal attenuation.

shown and bound by a colored box) is used to estimate the maximum OCT signal intensity profile versus physical depth, shown in Supplementary Fig. 6(b). In Supplementary Fig. 6(b), we overlaid the maximum axial OCT intensity, $a_{MIPxy}{}^2(z)$, with the square of the beam axial irradiance profiles, shown in Supplementary Fig. 1(e), accounting for the medium refractive index, *n*, as in the analytical model proposed in Bisaillon *et al.*[3] and Hillman[17], where the authors fit a Lorentzian function for a Gaussian beam. For the Bessel beam, we used and adapted the analytical expression of Lorenser *et al.*[9]:

$$a_{MIPxy}{}^{2}(z) \propto I_{beam}{}^{2}\left(z, z_{DOF}, z_f, n\right), \quad \text{(S2)}$$

$$I_G \propto \left(1+4\left(\frac{z-z_{f_G}}{z_{DOF_G}}\right)^2\right)^{-1}, \quad \text{(S2a)}$$

$$I_B \propto \frac{z-z_{f_B}}{z_{DOF_B}} \exp\left(-1.28\left(\frac{z-z_{f_B}}{z_{DOF_B}}\right)^2\right), \quad \text{(S2b)}$$

where I_G and I_B are the Gaussian and Bessel beam irradiances, respectively, $z_{DOF_G} = 2z_r = 2n \cdot \pi\omega_0{}^2 / \lambda_0$ is twice the Gaussian Rayleigh range in the medium, the Bessel DOF is $z_{DOF_B} \cong 1.26N \cdot z_{DOF_G}$, N is the Fresnel number, and z and z_f (the focus position in the medium) are physical lengths. We then displayed the analytical fits on a logarithmic intensity scale as $10 \cdot \log_{10} a_{MIPxy}{}^2(z)$. There is a good agreement between the experimental data and the analytical curves.

OCT B-scan images of the contrast phantom with both beams are presented in Supplementary Fig. 6(c). Depth profiles are averaged (on a linear basis) along a 100 μm-thick portion of the embedding casting in the areas highlighted in the figure by a coloured rectangle. The resulting signal should suffer only a small attenuation with depth, as the embedding casting has a scattering coefficient $\mu_s < 1$ mm^{-1}.

In the case of fully developed speckled signal arising from a multitude of scatterers being probed by the illumination beam, but still under a single scattering model[17], the transversally averaged linear OCT intensity, $\bar{a}(z)$, needs to be fitted[16] with the product of the confocal response (sometimes called the confocal axial PSF) and the exponential signal decay term. Unlike the case of a single scatterer being illuminated, described by Eq. (S2), the confocal response of the OCT signal intensity can be modelled by a beam axial irradiance profile with an apparent DOF corresponding to twice the beam DOF in the medium[15]. If we assume that the theory of van Leeuwen *et al.*[15] applies to both beam types, then:

$$\bar{a}^2(z) \propto I_{beam}(z, z_{DOFapp}, z_f, n) e^{-2\mu_t(\rho)z}, \quad \text{(S3)}$$

$$z_{DOFapp} = 2z_{DOF}, \quad \text{(S3a)}$$

where z_{DOFapp} is the apparent OCT signal DOF, and μ_t is the OCT attenuation coefficient, which depends on the scatterer concentration ρ.

The theoretical curves overlaid on the experimental curves are shown in Supplementary Fig. 6(d), displayed on a logarithmic intensity scale as $10 \cdot \log_{10} \overline{a}^2(z)$. The dotted curves at the bottom of the figure represent the exponential attenuation term, with an attenuation coefficient of less than 1 mm^{-1}. The curves are in very good agreement with the experimental data. The good agreement of the experimental data with OCT single scattering signal models for both phantoms validates the claim that our imaging targets do not introduce a significant amount of multiple scattering in the image and, as such, they are good scattering targets to probe the influence of the overlayers on image quality.

Note that in Supplementary Fig. 6(d), the maximum SNR penalty is more than 7.5dB. This is because, during the acquisition of the contrast phantom images, the two-beam sample-arm roundtrip losses were not equalized, unlike in the case of the PSF phantom images; thus, the roundtrip path loss of the Bessel beam is 4dB higher than that of the Gaussian beam.

Results

The simulated OCT PSF is obtained from the square of the simulated transverse illumination irradiance profiles [9,17,18]. Supplementary Fig. 7 shows, on a logarithmic scale, the simulated OCT transverse PSFs (Supplementary Fig. 7(a)) and the experimental PSFs (Supplementary Fig. 7(b)) for both beams, after propagation through the SF overlayer, Overlayer 2 and Overlayer 3 in two transverse planes, i.e., 140 µm before focus and at focus (top and bottom, respectively).

Supplementary Fig. 7 shows good qualitative agreement between the simulated and experimental transverse PSFs, compatible with the necessarily different realizations of the coherent (speckled) field between the two.

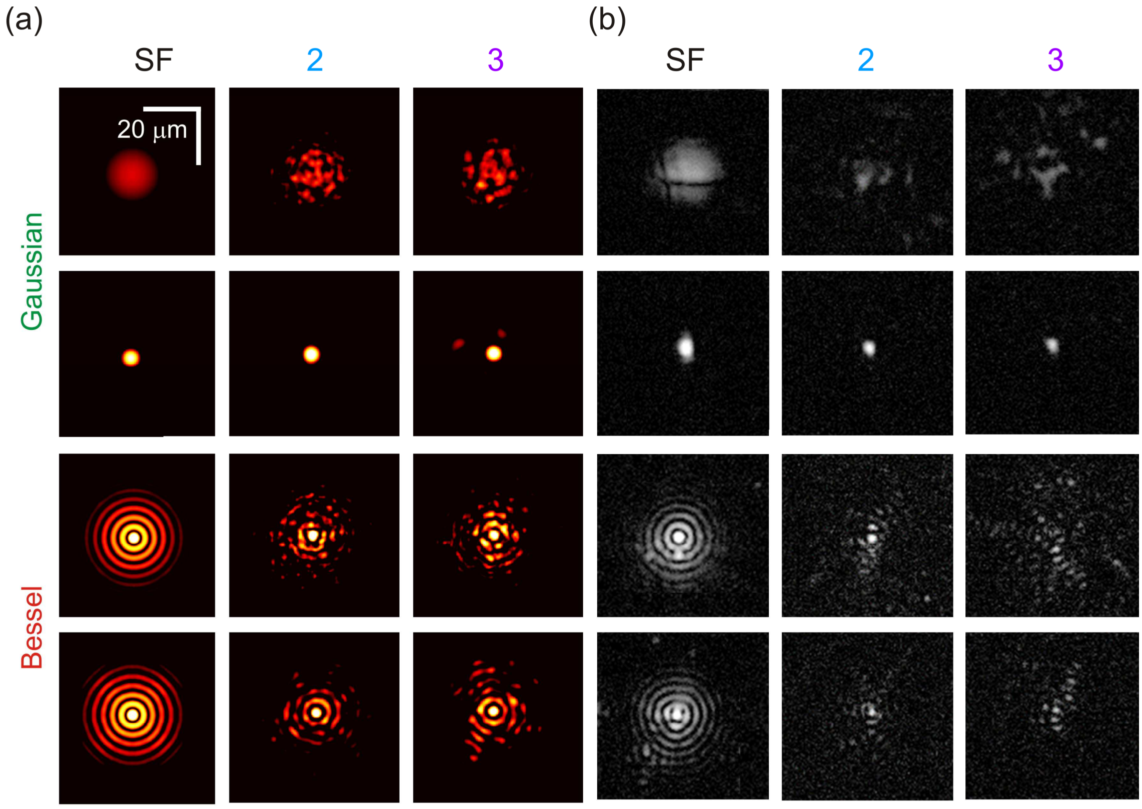

Supplementary Figure 7. (a) Simulated and (b) experimental transverse PSFs for Gaussian and Bessel beams, top and bottom, respectively. The top row for each beam is 140 μm before focus, and the bottom row is at focus. SF: Scattering-free overlayer; 2: Scattering overlayer 2; 3: Scattering overlayer 3. Dynamic ranges in the images are as follows: Gaussian SF: 40dB; Gaussian 2, 3: 30dB; Bessel SF: 30dB; Bessel 2, 3: 20dB.

Because of this agreement, and the additional independent validation of the electromagnetic numerical simulation through scattering media presented in [1,19], in the main paper, we present the signal-to-background ratio evaluated from the simulated beam irradiances.

Discussion

In the main paper, we focused our analysis on changes in OCT contrast, i.e., the relative ratio of OCT SNRs between neighbouring regions of a sample, rather than the changes in absolute OCT SNRs in a given region of the sample. This means that we could tolerate different insertion losses between the Gaussian and Bessel beam sample arms in the experimental OCT setup, as these differences only affect the absolute SNRs of the images.

The 3D structured phantom proved invaluable in thoroughly benchmarking OCT contrast between Gaussian and Bessel beams. Nevertheless, some observations regarding experimental uncertainties should be made here. Manufacturing tolerances may have resulted in a difference in concentrations between the pillars and the embedding casting slightly larger than the design value. This could be the reason why we observe more than 10 dB pillar-to-embedding casting contrast in the images. Also, as the concentration of TiO_2 in the embedding casting is very low, in some instances areas of non-fully developed speckles and in others areas absent of signal are observed in the Gaussian images around the focus. This added variability in the low signal is the reason why the speckle contrast ratio[18] seems higher in some places outside the pillars than within them. As the embedding casting speckle pattern appears more “noisy” in the Gaussian beam image than in the Bessel beam image, this creates an issue known as low contrast resolution[20] which could also be the reason for the apparent invisibility of the 10 μm pillar in the Gaussian beam image, otherwise visible in the Bessel beam image.

In our rigorous EM simulations, we evaluated the signal-to-background ratio only on the optical axis. However, this is a simplification of the relative contribution of image-forming and image-degrading contributions to an OCT image in a dense turbid tissue sample. To draw a quantitative link between the signal-to-background ratios and the OCT contrast degradation magnitude, a full OCT PSF simulation (including both illumination, detection and transverse scanning) would be required. In such a simulation, the effect of multiple scattering on the total collected field should be evaluated for each transverse position. This could be done by calculating the integral over the collection fibre between the scalar product of the illumination field and the field backscattered by the specific scattering potential probed by the illumination field, as a function of wavenumber, but this calculation is beyond the scope of this paper. A full 3D OCT simulation will allow us to quantify this effect in a rigorous manner and work is underway in this direction[19].

Despite that, our current analysis of the SBR already shows that the Gaussian beam outperforms the Bessel beam in terms of retaining native contrast for equal parameters at focus.

References

1. Török, P., Munro, P.R.T. & Kriezis, E.E. High numerical aperture vectorial imaging in coherent optical microscopes. *Opt. Express* **16**, 507-523 (2008).
2. Garces-Chavez, V., McGloin, D., Melville, H., Sibbett, W. & Dholakia, K. Simultaneous micromanipulation in multiple planes using a self-reconstructing light beam. *Nature* **419**, 145-147 (2002).
3. Bisaillon, C.E., Lamouche, G., Maciejko, R., Dufour, M. & Monchalin, J.P. Deformable and durable phantoms with controlled density of scatterers. *Phys. Med. Biol.* **53**, N237-N247 (2008).
4. Woolliams, P.D., Ferguson, R.A., Hart, C., Grimwood, A. & Tomlins, P.H. Spatially deconvolved optical coherence tomography. *Appl. Opt.* **49**, 2014-2021 (2010).
5. Liu, Q.H. The PSTD algorithm: A time-domain method requiring only two cells per wavelength. *Microw. Opt. Technol. Lett.* **15**, 158-165 (1997).
6. Taflove, A. & Hagness, S. *Computational Electrodynamics: The Finite-Difference Time-Domain Method, Third Edition*, (Artech House, 2005).
7. Munro, P.R.T., Engelke, D. & Sampson, D.D. A compact source condition for modelling focused fields using the pseudospectral time-domain method. *Opt. Express* **22**, 5599-5613 (2014).
8. Matsushima, K. & Shimobaba, T. Band-limited angular spectrum method for numerical simulation of free-space propagation in far and near fields. *Opt. Express* **17**, 19662-19673 (2009).
9. Lorenser, D., Christian Singe, C., Curatolo, A. & Sampson, D.D. Energy-efficient low-Fresnel-number Bessel beams and their application in optical coherence tomography. *Opt. Lett.* **39**, 548-551 (2014).

10. Bohren, C.F. & Huffman, D.R. *Absorption and Scattering of Light by Small Particles*, (Wiley-VCH Verlag GmbH, 2007).
11. Prahl, S.A., *Mie scattering calculator*. (2006) Available at: http://www.omlc.ogi.edu/calc/miecalc.html.
12. Woolliams, P.D. & Tomlins, P.H. The modulation transfer function of an optical coherence tomography imaging system in turbid media. *Phys. Med. Biol.* **56**, 2855 (2011).
13. Scolaro, L., *et al.* Parametric imaging of the local attenuation coefficient in human axillary lymph nodes assessed using optical coherence tomography. *Biomed. Opt. Express* **3**, 366-379 (2012).
14. Kalkman, J., Bykov, A.V., Streekstra, G.J. & van Leeuwen, T.G. Multiple scattering effects in Doppler optical coherence tomography of flowing blood. *Phys. Med. Biol.* **57**, 1907 (2012).
15. van Leeuwen, T.G., Faber, D.J. & Aalders, M.C. Measurement of the axial point spread function in scattering media using single-mode fiber-based optical coherence tomography. *IEEE J. Sel. Top. Quantum Electron.* **9**, 227-233 (2003).
16. Faber, D.J., van der Meer, F.J., Aalders, M.C. & van Leeuwen, T.G. Quantitative measurement of attenuation coefficients of weakly scattering media using optical coherence tomography. *Opt. Express* **12**, 4353-4365 (2004).
17. Hillman, T.R. *Microstructural Information beyond the Resolution Limit: Studies in Two Coherent, Wide-field Biomedical Imaging Systems* (The University of Western Australia, School of Eletrical, Electronic and Computer Enginnering, 2007).
18. Curatolo, A., Kennedy, B.F., Sampson, D.D. & Hillman, T.R. Speckle in Optical Coherence Tomography. in *Advanced Biophotonics: Tissue Optical Sectioning,* 211-277 (Taylor & Francis, 2014).

19. Munro, P.R.T., Curatolo, A. & Sampson, D.D. Full wave model of image formation in optical coherence tomography applicable to general samples. *Opt. Express* **23**, 2541-2556 (2015).

20. Baker, M. E., *et al.* Contrast-to-noise ratio and low-contrast object resolution on full- and low-dose MDCT: SAFIRE versus filtered back projection in a low-contrast object phantom and in the liver, *Am. J. Roentgenol.* **199**, 8-18 (2012).
